# Supplementary material for: Robust dynamic community detection with applications to human brain functional networks
Source: Nat Commun. 2020 Jun 5;11:2785. doi: 10.1038/s41467-020-16285-7 (PMC7275079; doi:10.1038/s41467-020-16285-7)
Supplement: Supplementary file 1 — Supplementary Information [file 41467_2020_16285_MOESM1_ESM.pdf]

Robust dynamic community detection with applications to human brain functional networks

Martinet et al.

Supplementary Methods

Pseudo-code for the *Plex* function

This function extracts all maximal k-plexes (adapted from [56]). Note this function is recursive. For simplicity, we refer to integer indices and vertices interchangeably in this pseudo-code.

MP ← Plex(*A, R, P, X, m, k*)

Input:

    A, $p \times p$ adjacency matrix representing a graph *G(V,E)*

    R, represents a currently growing k-plex (initially empty)

    P, prospective vertices that may be added to the growing plex in *R* (initially {1,2,...,*p*})

    X, vertices already processed during the recursive algorithm's execution (initially empty)

    m, minimum k-plex size

    k, k-plex parameter

Output:

    MP, list of maximal k-plexes found in *A*, represented as sets of vertices (integers)

If *P* empty and *X* empty and $|R| > k$ **then**

    MP ← {*R*}

Else

    For each vertex *u* in *P* **do**

        Rnew ← *R* U {*u*}

        P ← *P* \ {*u*}

        Nu ← {vertex *v* in *V* | deg(*v* in subgraph Rnew U {*v*}) > |Rnew| − *k*}

        Crit ← {vertex v in Rnew | deg(*v* in subgraph Rnew) = |Rnew| − *k*}

        If |Crit| > 0 **then**

            Nu ← Nu ∩ {$v \in V$ | $\forall c \in$ Crit, $A(c,v) = 1$}

End if

MP ← MP U **Plex**(*A*, Rnew, *P* ∩ Nu, *X* ∩ Nu, *m*, *k*)

X ← *X* U {*u*}

End for

End if

Pseudo-code for the *StatComm* function

This function is used to detect static communities from the set of k-plexes identified by the *Plex* function.

B ← **StatComm(*A, m, k*)**

Input:

A, *p* x *p* adjacency matrix

m, minimum k-plex size

k, k-plex parameter

Output:

B, *p* x *p* matrix of label sets such that *B(i,j)* is the set of communities edge (*i*,*j*) belongs to.

MP ← **Plex**(*A*, {}, {1,2,...,*p*}, {}, *m*, *k*)

*G** ← Graph(*V**,*E**) such that *V** = MP and *E**(*i*,*j*) = 1 if |MPi ∩ MPj| ≥ *m*−1

*C**1, *C**2,..., *C**o ← Connected Components of *G** (e.g., computed via breadth-first search)

For every *l* = 1,2,...,o **do**

For every k-plex MPj ∈ Cl∗ **do**

For each vertex vi ∈ MPj **do**

For each vertex vh ∈ MPj with vh > vi **do**

If *A*(*i*,*h*) = 1 **then**

```
            B(i,h) ← B(i,h) U {l}
          End if
        End for
      End for
    End for
End for
```

Pseudo-code for the *DynComm* function

This function detects dynamic communities between two subsequent graphs by building an enhanced adjacency matrix as an input for the *StatComm* function.

```
C ← DynComm(A1, A2, m, k)
Input:
  A1, A2, p × p adjacency matrices
  m, minimum k-plex size
  k, k-plex parameter
Output:
  C, a p × p matrix of sets of integers
A ← 2p x 2p matrix [A1    Id(p)]
                   [Id(p)    A2] where Id(p) is the square identity matrix of size p
For every i = 1, 2, ..., p − 1 do
  For every j = (i + 1), ..., p do
    If A1(i,j) = 1 and A2(i,j) = 1 then
      A(i,p+j) ← 1
      A(j,p+1) ← 1
```

A(p+j,i) ← 1

A(p+i,j) ← 1

End if

End for

End for

C ← **StatComm**(A, m, k)

Supplementary Tables

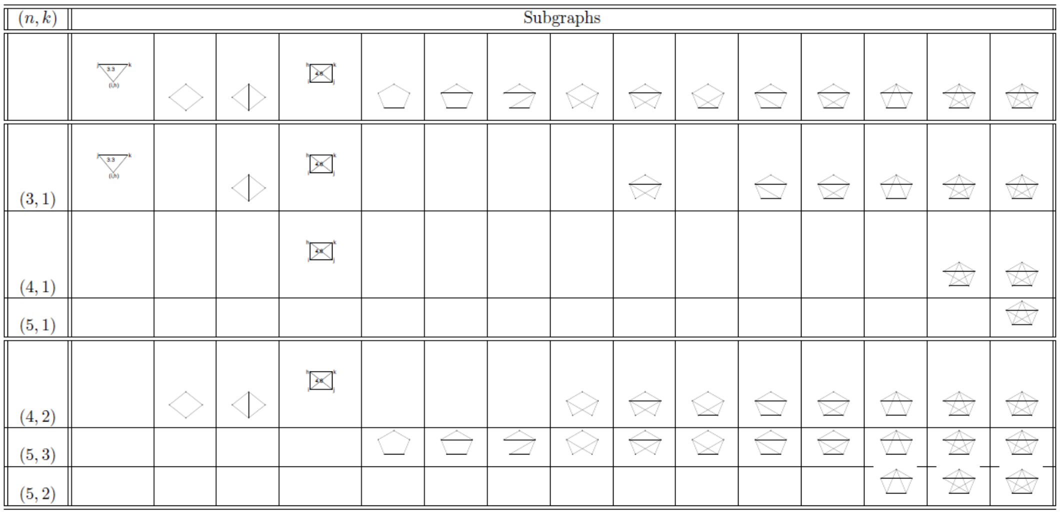

Supplementary Table 1: Network motifs detectable via plexes. We display all subgraphs on *m*=3, 4, or 5 vertices which can be discovered by order *k*=1, 2, and 3 plexes. The top row represents the motifs which we seek to discover and which will form the themes as an aggregation thereof. The next six rows represent the order (*m,k*) plex which can discover such a motif in the respective column.

Supplementary Figures

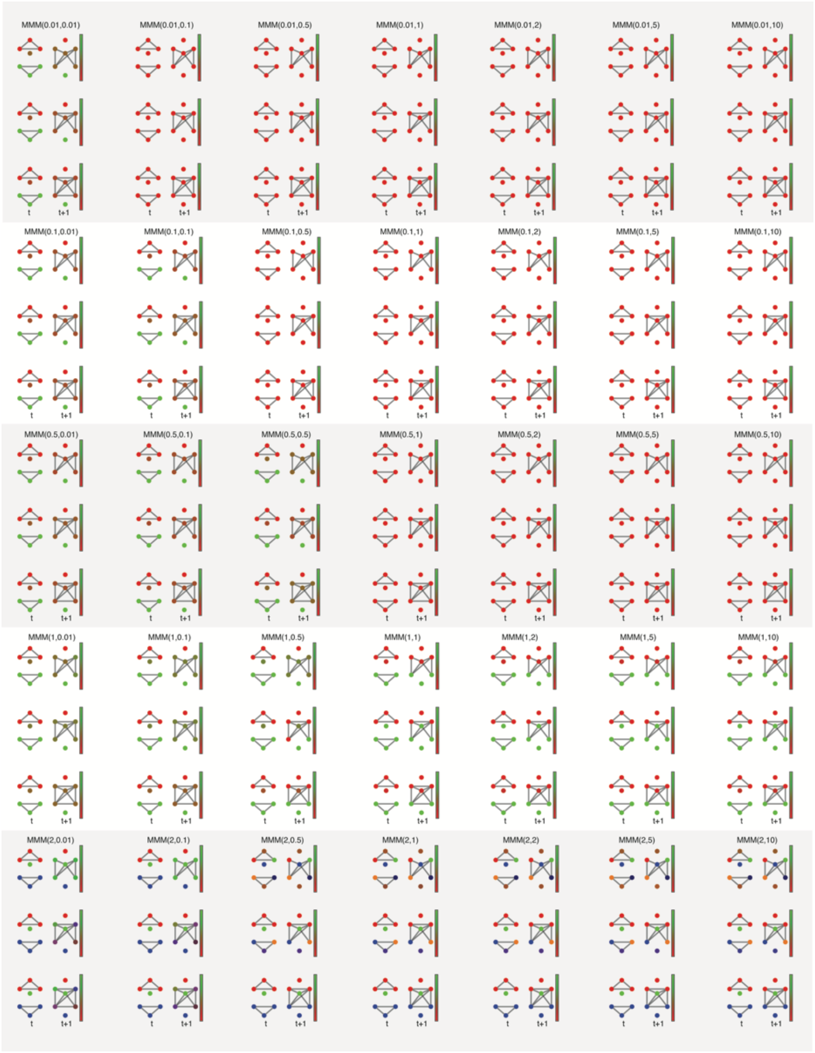

Supplementary Figure 1. Comparison of MMM computed with different parameter configurations. We apply MMM using all combinations of γ ={0.01, 0.1, 0.5, 1, 2} and ω = {0.01, 0.1, 0.5, 1, 2, 5, 10} to determine communities across two adjacent time points t and $t+1$. The connected component at time $t+1$ shares increasingly more edges from the top to the bottom row of each subfigure, as in Figure 1. No combination of gamma and omega shown here distinguishes the three cases. The colorbar indicates the proportion of community membership over 100 repetitions of community detection. The titles MMM(γ, ω) indicate the values of parameters γ and ω.

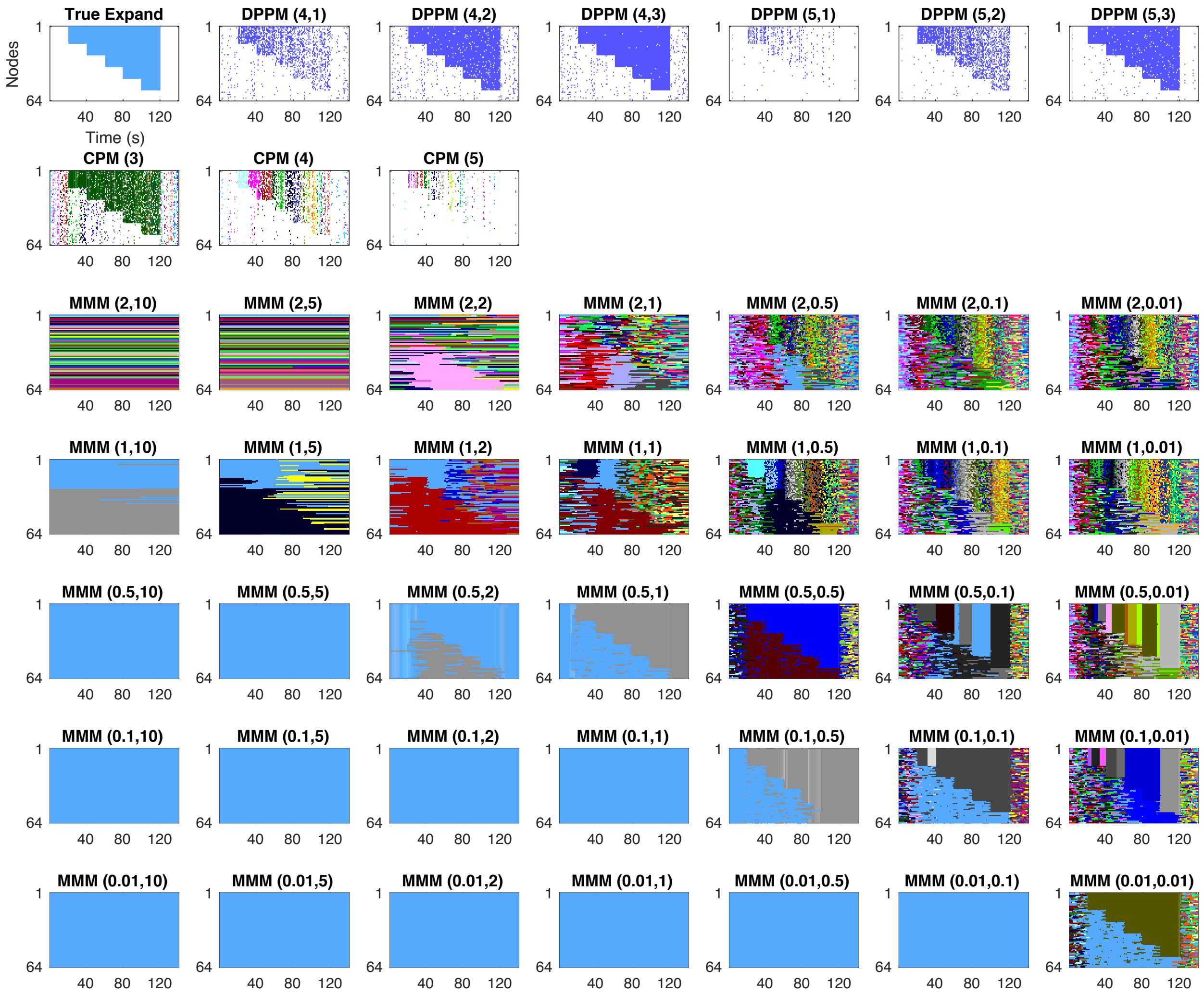

Supplementary Figure 2: Example dynamic community detections applied to a simulation of community expansion. For each method, we consider the following parameter configurations: DPPM with (m,k) equal to (4,1), (4,2), (4,3), (5,1), (5,2) and (5,3); CPM with m equal to (3,4,5); MMM with all combinations of γ = {0.01,0.1,0.5,1,2}, ω = {0.01,0.1,0.5,1,2,5,10}. The true expansion is illustrated in the upper left subfigure. Color indicates community membership. The largest community detected by DPPM is most consistent with the true expansion.

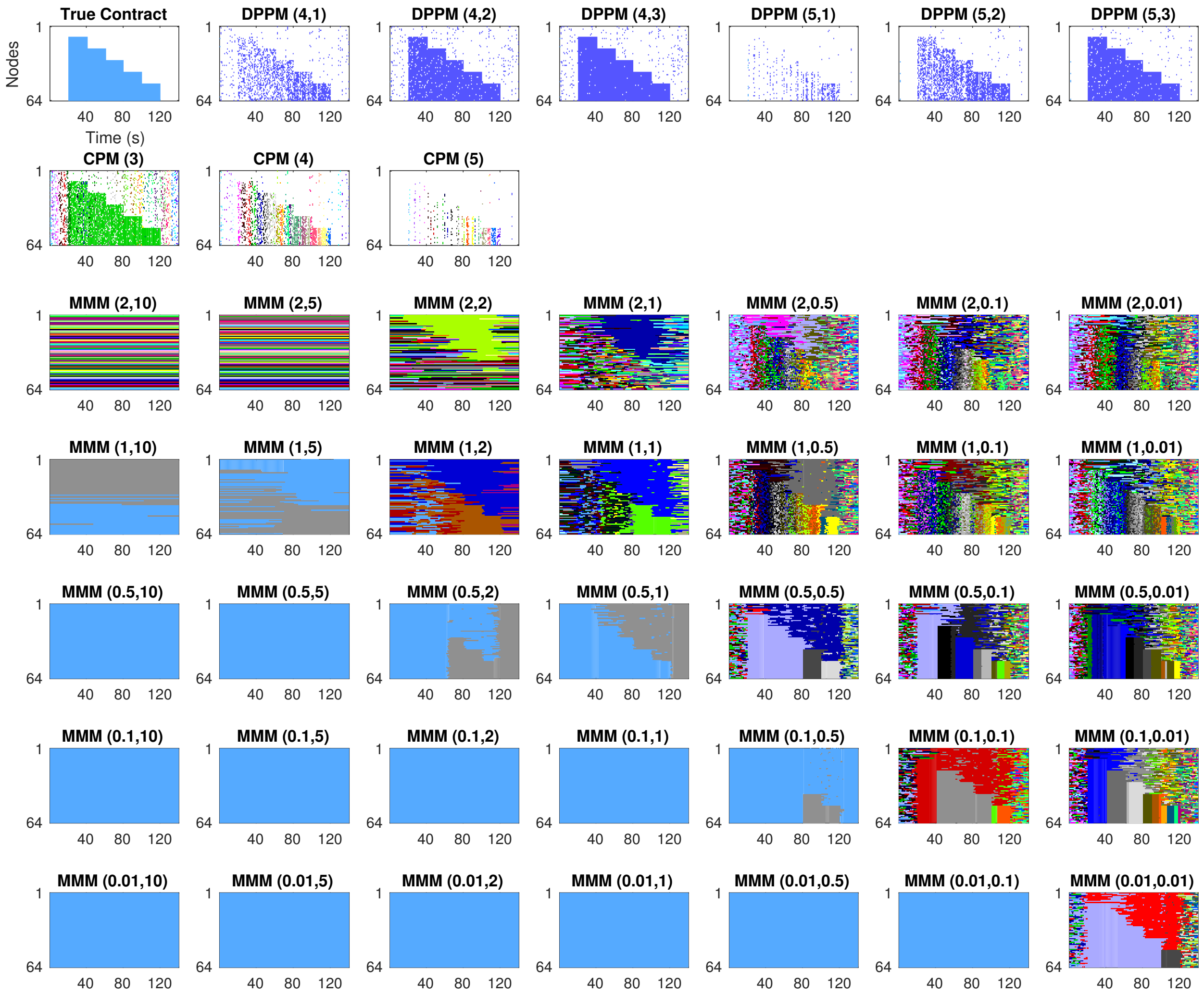

Supplementary Figure 3: Example dynamic community detections applied to a simulation of community contraction. For each method, we consider the parameter configurations listed in the caption of Supplementary Figure 2. The true contraction is illustrated in the upper left subfigure. Color indicates community membership. The largest community detected by DPPM is most consistent with the true contraction.

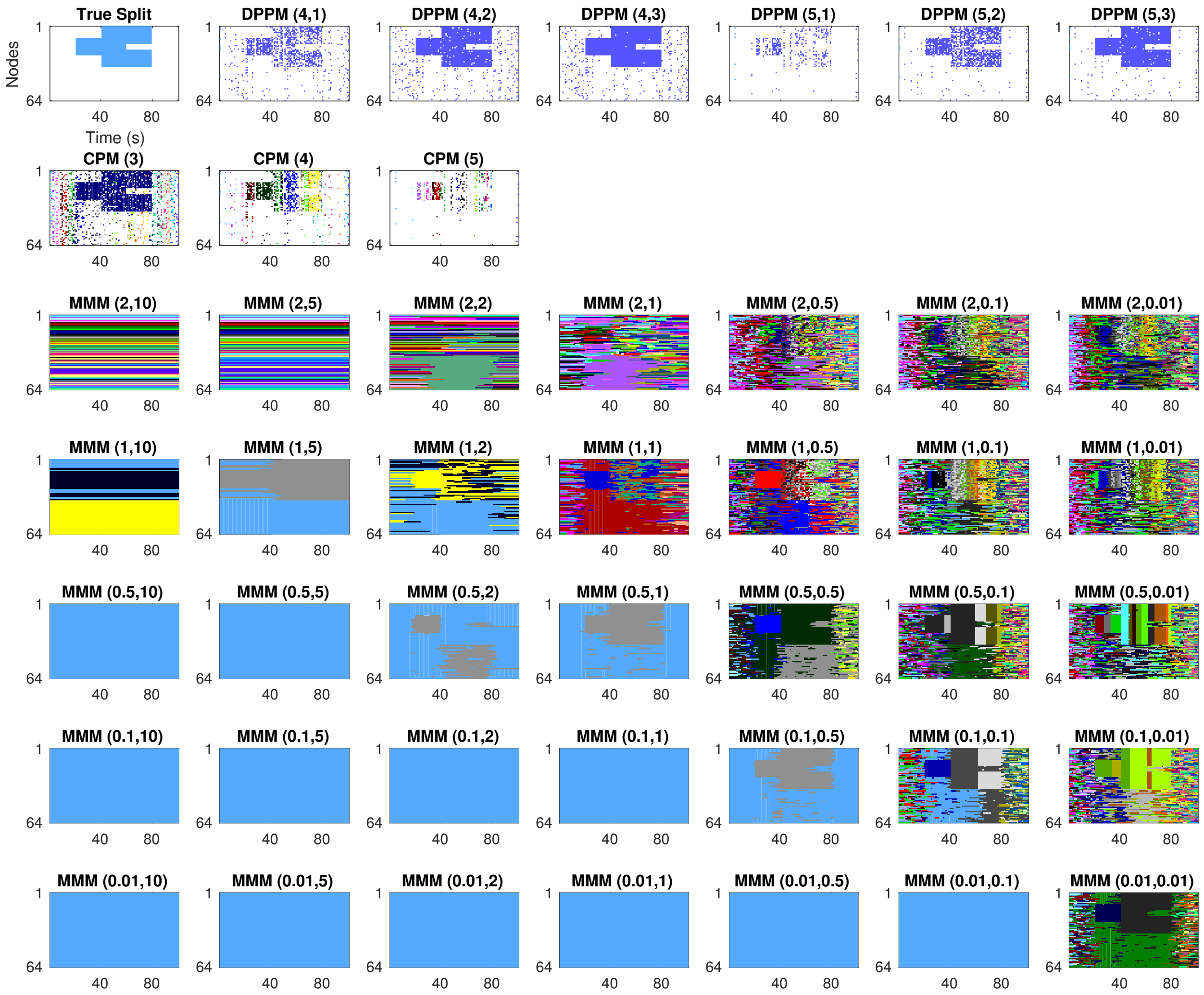

Supplementary Figure 4: Example dynamic community detections applied to a simulation of a community splitting. For each method, we consider the parameter settings listed in the caption of Supplementary Figure 2. The true splitting is illustrated in the upper left subfigure. Color indicates community membership. The largest community detected by DPPM is most consistent with the true community split.

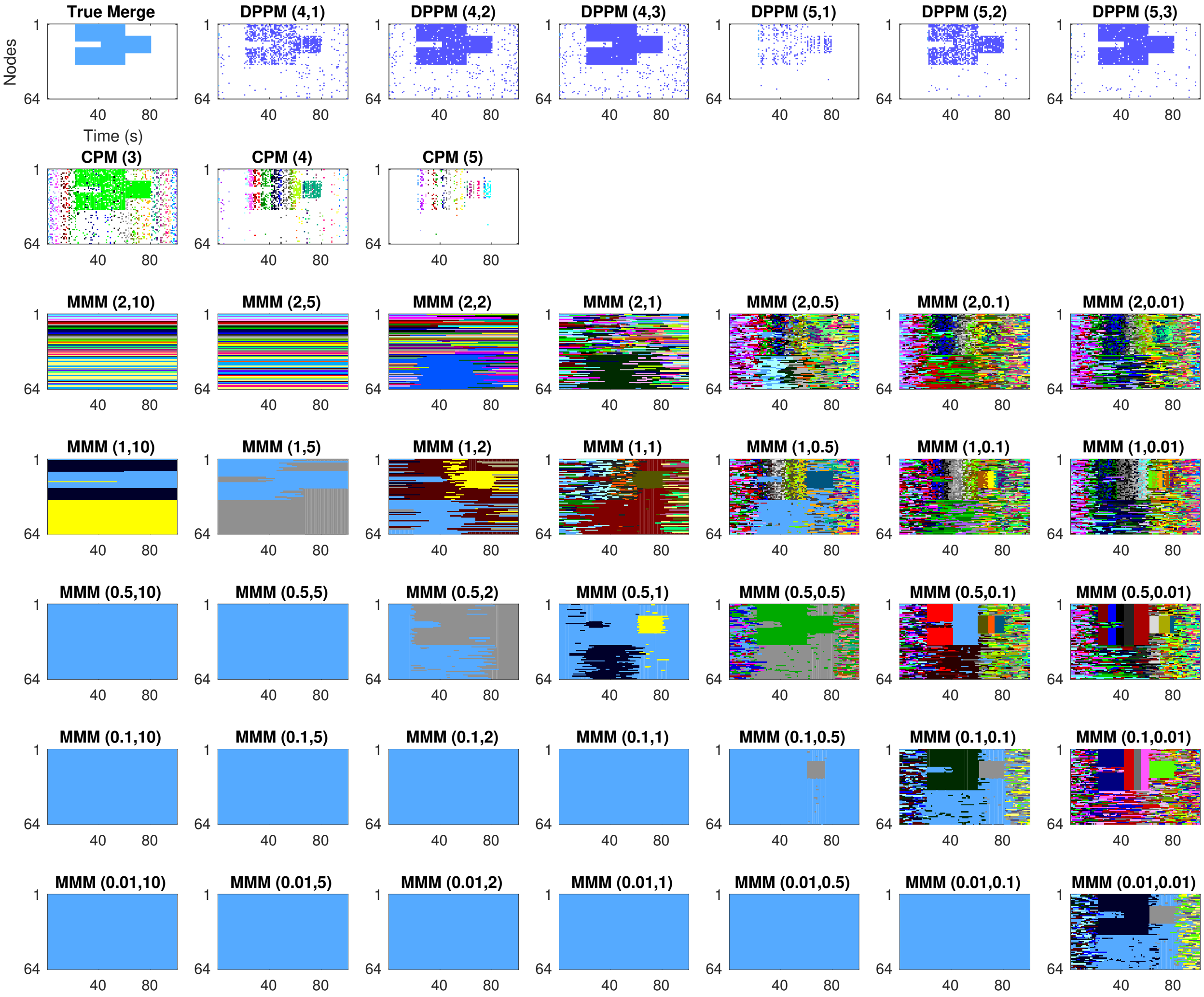

Supplementary Figure 5: Example dynamic community detections applied to a simulation of a community merging. For each method, we consider the parameter settings listed in the caption of Supplementary Figure 2. The true merging is illustrated in the upper left subfigure. Color indicates community membership. The largest community detected by DPPM is most consistent with the true community merge.

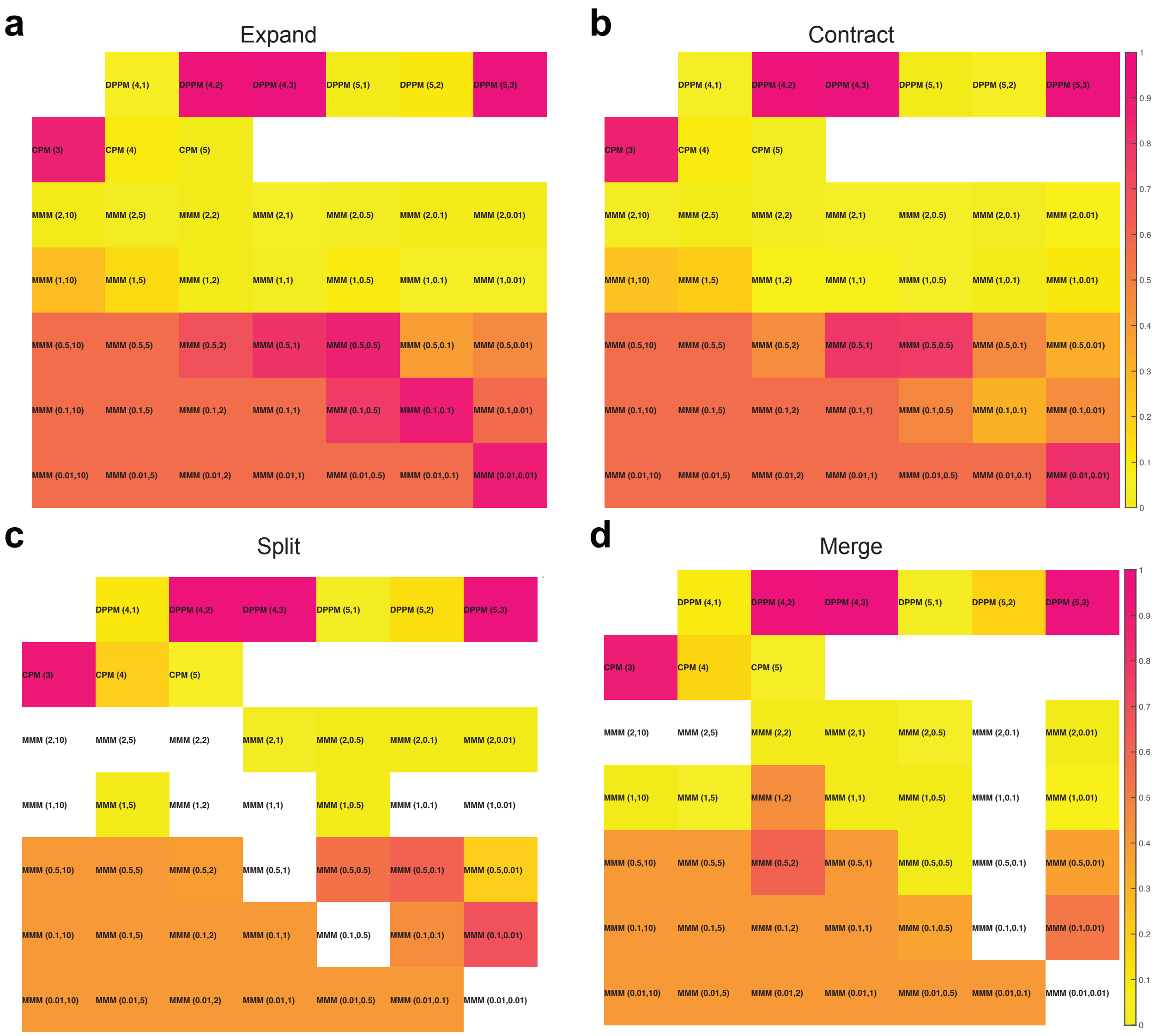

Supplementary Figure 6: Illustration of performance of different parameter regimes. The F_1 score computed for each example simulation shown in Supplementary Figures 2-5: **(a)** Expand in Supplementary Figure 2, **(b)** Contract in Supplementary Figure 3, **(c)** Split in Supplementary Figure 4, **(d)** Merge in Supplementary Figure 5. For each method, we consider the parameter settings listed in the caption of Supplementary Figure 2. The 7-by-7 grid in each subfigure matches the layout of parameter settings and methods shown in Supplementary Figures 2-5; see also text at each grid point. Dark (light) colors indicate large (small) F_1 score; colorbars at right.

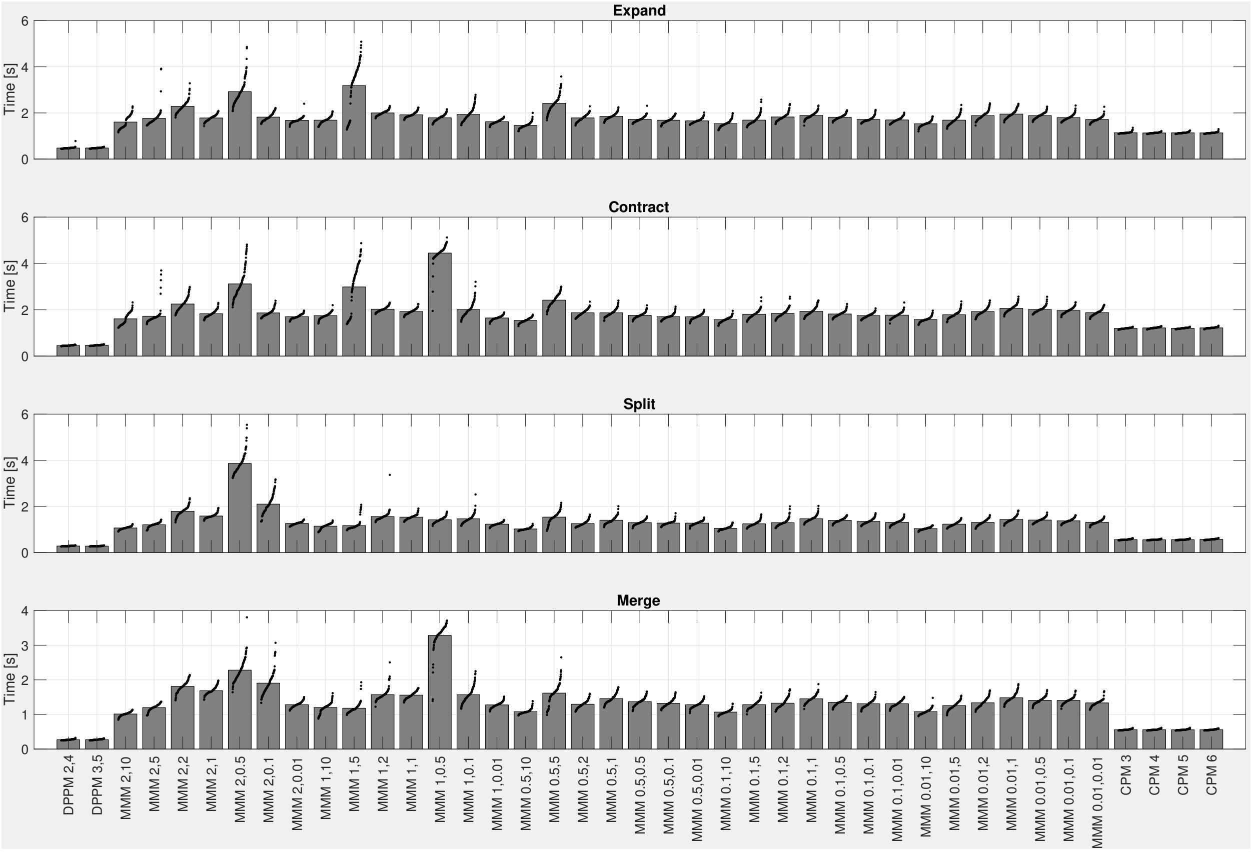

Supplementary Figure 7: DPPM tends to run in less time than MMM and CPM. Run times for each community detection method applied to each simulation scenario. Each simulation scenario (row) shows the means (bar) of the run times calculated for each method (columns) for n=100 independent realizations of the simulated dynamic networks with different noise instantiations. Dots indicate the results for each of the n=100 simulations for each simulation scenario and method. The bottom subfigure lists the community detection method and the parameter configuration. The parameter configurations include DPPM with (m,k) equal to {(4,2), (5,3)}; MMM with all 35 combinations of γ = {0.01,0.1,0.5,1,2}, ω = {0.01,0.1,0.5,1,2,5,10}; CPM with m equal to {3,4,5,6}.
