# Supplementary material for: Robust dynamic community detection with applications to human brain functional networks
Source: Nat Commun. 2020 Jun 5;11:2785. doi: 10.1038/s41467-020-16285-7 (PMC7275079; doi:10.1038/s41467-020-16285-7)
Supplement: Supplementary file 3 — Reporting Summary [file 41467_2020_16285_MOESM3_ESM.pdf]

nature research

Corresponding author(s): Eric Kolaczyk

Last updated by author(s): Mar 13, 2020

Reporting Summary

Nature Research wishes to improve the reproducibility of the work that we publish. This form provides structure for consistency and transparency in reporting. For further information on Nature Research policies, see Authors & Referees and the Editorial Policy Checklist .

Statistics

For all statistical analyses, confirm that the following items are present in the figure legend, table legend, main text, or Methods section.

n/a	Confirmed	
☐	☒	The exact sample size (*n*) for each experimental group/condition, given as a discrete number and unit of measurement
☐	☒	A statement on whether measurements were taken from distinct samples or whether the same sample was measured repeatedly
☐	☒	The statistical test(s) used AND whether they are one- or two-sided<br>*Only common tests should be described solely by name; describe more complex techniques in the Methods section.*
☐	☒	A description of all covariates tested
☒	☐	A description of any assumptions or corrections, such as tests of normality and adjustment for multiple comparisons
☐	☒	A full description of the statistical parameters including central tendency (e.g. means) or other basic estimates (e.g. regression coefficient) AND variation (e.g. standard deviation) or associated estimates of uncertainty (e.g. confidence intervals)
☐	☒	For null hypothesis testing, the test statistic (e.g. *F*, *t*, *r*) with confidence intervals, effect sizes, degrees of freedom and *P* value noted<br>*Give P values as exact values whenever suitable.*
☒	☐	For Bayesian analysis, information on the choice of priors and Markov chain Monte Carlo settings
☒	☐	For hierarchical and complex designs, identification of the appropriate level for tests and full reporting of outcomes
☐	☒	Estimates of effect sizes (e.g. Cohen's *d*, Pearson's *r*), indicating how they were calculated

Our web collection on statistics for biologists contains articles on many of the points above.

Software and code

Policy information about availability of computer code

Data collection	Dynamic network models were simulated using the algorithm defined here: https://github.com/MultilayerGM/MultilayerGM-MATLAB , Version 1.0.1. Clinical data from an existing study were used as an example application.
Data analysis	All analyses and modeling were performed using custom designed algorithms written in MATLAB (MathWorks, Inc). A complete implementation of the dynamic plex percolation method (including the subroutines Plex, StatComm, and DynComm) is available for re-use and further development at the repository: https://github.com/Eden-Kramer-Lab/dppm .<br>The multilayer modularity method (including the GenLouvain function) are available at: http://netwiki.amath.unc.edu/GenLouvain/GenLouvain , Version 2.1

For manuscripts utilizing custom algorithms or software that are central to the research but not yet described in published literature, software must be made available to editors/reviewers. We strongly encourage code deposition in a community repository (e.g. GitHub). See the Nature Research guidelines for submitting code & software for further information.

Data

Policy information about availability of data

All manuscripts must include a data availability statement. This statement should provide the following information, where applicable:

- Accession codes, unique identifiers, or web links for publicly available datasets
- A list of figures that have associated raw data
- A description of any restrictions on data availability

The seizure data that support the findings of this study are available on request from the authors. The data are not publicly available due to them containing information that could compromise research participant privacy/consent.

Field-specific reporting

Please select the one below that is the best fit for your research. If you are not sure, read the appropriate sections before making your selection.

☒ Life sciences ☐ Behavioural & social sciences ☐ Ecological, evolutionary & environmental sciences

For a reference copy of the document with all sections, see nature.com/documents/nr-reporting-summary-flat.pdf

Life sciences study design

All studies must disclose on these points even when the disclosure is negative.

Sample size	No sample-size calculation was performed. Existing human subject data (Figure 5) were analyzed to illustrate method performance, and the clinical results should be considered preliminary. Using the estimated means and standard deviations from the results in Figure 5, we expect that the test for the number of communities is underpowered (power of approximately 60%) while the test for the duration of the longest community is sufficiently powered (power over 80%). However, we note that these calculations do not account for the dependence of samples; if a clinical study is pursued, we encourage a more detailed analysis of sufficient sample size.
Data exclusions	No data were excluded from the study.
Replication	Dynamic network simulations were repeated 100 times, and the mean values were reported. These replications (Figure 4) confirmed the example results (Figure 2,3). The results of each simulation for all replications are shown in Figure 4.
Randomization	Experimental groups were defined in the dynamic network simulations ("expand", "contract", "split", and "merge") to mimic dynamic network features expected to occur in vivo.
Blinding	Blinding was not possible in this study. The goal was to propose a new dynamic network analysis method, and compare its performance to two existing methods.

Reporting for specific materials, systems and methods

We require information from authors about some types of materials, experimental systems and methods used in many studies. Here, indicate whether each material, system or method listed is relevant to your study. If you are not sure if a list item applies to your research, read the appropriate section before selecting a response.

Materials & experimental systems

n/a	Involved in the study	
☒	☐	Antibodies
☒	☐	Eukaryotic cell lines
☒	☐	Palaeontology
☒	☐	Animals and other organisms
☐	☒	Human research participants
☒	☐	Clinical data

Methods

n/a	Involved in the study	
☒	☐	ChIP-seq
☒	☐	Flow cytometry
☒	☐	MRI-based neuroimaging

Human research participants

Policy information about studies involving human research participants

Population characteristics	We utilized invasive brain voltage data recorded from 12 patients with epilepsy. These data were previously collected as part of standard clinical care and analyzed in an existing study. Please see Table 1 of [Martinet et al, J Neurosci, 35(25):9477–9490, 2015] for detailed patient information, including age and gender.
Recruitment	Patients are referred to the epilepsy program for possible surgical treatment of epilepsy without regard to race, ethnic origin, or gender. Thus, men and women, and all racial and ethnic groups, are represented and in the study population approximately in proportion to their representation in the general population.
Ethics oversight	All human subjects were enrolled after informed consent was obtained and approval was granted by local Institutional Review Boards at Massachusetts General Hospital and at Boston University according to National Institutes of Health guidelines.

Note that full information on the approval of the study protocol must also be provided in the manuscript.
